# Platelet-to-White Blood Cell Ratio Is Associated with Adverse Outcomes in Cirrhotic Patients with Acute Deterioration

**DOI:** 10.3390/jcm11092463

**Published:** 2022-04-27

**Authors:** Jung-Hee Kim, Sung-Eun Kim, Do-Seon Song, Hee-Yeon Kim, Eileen L. Yoon, Tae-Hyung Kim, Young-Kul Jung, Ki-Tae Suk, Baek-Gyu Jun, Hyung-Joon Yim, Jung-Hyun Kwon, Sung-Won Lee, Seong-Hee Kang, Moon-Young Kim, Soung-Won Jeong, Jae-Young Jang, Jeong-Ju Yoo, Sang-Gyune Kim, Young-Joo Jin, Gab-Jin Cheon, Byung-Seok Kim, Yeon-Seok Seo, Hyung-Su Kim, Dong-Hyun Sinn, Woo-Jin Chung, Hwi-Young Kim, Han-Ah Lee, Seung-Woo Nam, In-Hee Kim, Jung-Il Suh, Ji-Hoon Kim, Hee-Bok Chae, Joo-Hyun Sohn, Ju-Yeon Cho, Yoon-Jun Kim, Jin-Mo Yang, Jung-Gil Park, Won Kim, Hyun-Chin Cho, Dong-Joon Kim

**Affiliations:** 1 Department of Internal Medicine, Hallym University College of Medicine, Chuncheon 24252, Korea; jungheekim@hallym.or.kr (J.-H.K.); ktsuk@hallym.ac.kr (K.-T.S.); hskim@kdh.or.kr (H.-S.K.); djkim@hallym.ac.kr (D.-J.K.); 2 Institute for Liver and Digestive Diseases, Hallym University, Chuncheon 24252, Korea; 3 Department of Internal Medicine, College of Medicine, The Catholic University of Korea, Seoul 06591, Korea; dsman@catholic.ac.kr (D.-S.S.); hee82@catholic.ac.kr (H.-Y.K.); doctorkwon@catholic.ac.kr (J.-H.K.); zambrotta@catholic.ac.kr (S.-W.L.); jmyangdr@catholic.ac.kr (J.-M.Y.); 4 Department of Internal Medicine, Hanyang University College of Medicine, Seoul 04763, Korea; mseileen80@hanyang.ac.kr (E.L.Y.); sonjh@hanyang.ac.kr (J.-H.S.); 5 Department of Internal Medicine, Korea University Ansan Hospital, Ansan 15355, Korea; lacid@korea.ac.kr (T.-H.K.); 93cool@hanmail.net (Y.-K.J.); gudwns21@korea.ac.kr (H.-J.Y.); drseo@korea.ac.kr (Y.-S.S.); kjhhepar@naver.com (J.-H.K.); 6 Department of Internal Medicine, Inje University Sanggye Paik Hospital, Seoul 01757, Korea; backyou78@hanmail.net (B.-G.J.); shkang14@yonsei.ac.kr (S.-H.K.); 7 Department of Internal Medicine, Yonsei University Wonju College of Medicine, Wonju 26426, Korea; drkimmy@yonsei.ac.kr; 8 Department of Internal Medicine, Soonchunhyang University College of Medicine, Seoul 04401, Korea; jeongsw@schmc.ac.kr (S.-W.J.); jyjang@schmc.ac.kr (J.-Y.J.); 9 Department of Internal Medicine, Soonchunhyang University Bucheon Hospital, Bucheon 14584, Korea; puby17@naver.com (J.-J.Y.); mcnulty@schmc.ac.kr (S.-G.K.); 10 Department of Internal Medicine, Inha University Hospital, Inha University School of Medicine, Incheon 22212, Korea; jyj412@hanmail.net; 11 Department of Internal Medicine, Gangneung Asan Hospital, University of Ulsan College of Medicine, Gangneung 25440, Korea; 1000@gnah.co.kr; 12 Department of Internal Medicine, Daegu Catholic University School of Medicine, Daegu 42472, Korea; kbs9225@cu.ac.kr; 13 Department of Medicine, Samsung Medical Center, Sungkyunkwan University School of Medicine, Seoul 06531, Korea; sinndhn@hanmail.net; 14 Department of Internal Medicine, Keimyung University School of Medicine, Daegu 42601, Korea; chung50@dsmc.or.kr; 15 Department of Internal Medicine, College of Medicine, Ewha Womans University, Seoul 07804, Korea; hwiyoung@gmail.com (H.-Y.K.); amelia86@naver.com (H.-A.L.); 16 Department of Internal Medicine, National Medical Center, Seoul 04564, Korea; kn37503@hotmail.com; 17 Department of Internal Medicine, Chonbuk National University Hospital, Chonbuk National University Medical School, Jeonju 54896, Korea; ihkimmd@chonbuk.ac.kr; 18 Department of Gastroenterology, Dongguk University College of Medicine, Kyongju 38067, Korea; sujungil@dongguk.ac.kr; 19 Department of Internal Medicine, Medical Research Institute, Chungbuk National University College of Medicine, Cheongju 28644, Korea; hbchae@chungbuk.ac.kr; 20 Department of Internal Medicine, College of Medicine, Chosun University, Gwangju 61452, Korea; jy.cho@chosun.ac.kr; 21 Department of Internal Medicine, Liver Research Institute, Seoul National University College of Medicine, Seoul 03080, Korea; yoonjun@snu.ac.kr; 22 Department of Internal Medicine, Yeungnam University College of Medicine, Daegu 42415, Korea; gsnrs@naver.com; 23 Department of Internal Medicine, Seoul Metropolitan Government Seoul National University Boramae Medical Center, Seoul 07061, Korea; wonshiri@yahoo.com; 24 Department of Internal Medicine, Gyeongsang National University Hospital, Jinju 52727, Korea; academic77@naver.com

**Keywords:** platelet-to-white blood cell ratio, acute-on-chronic liver failure, liver cirrhosis, acute decompensation, adverse outcomes

## Abstract

Background: The platelet-to-white blood cell ratio (PWR) is a hematologic marker of the systemic inflammatory response. Recently, the PWR was revealed to have a role as an independent prognostic factor for mortality in patients with hepatitis B virus (HBV)-related acute-on-chronic failure (ACLF) and HBV-related liver cirrhosis (LC) with acute decompensation (AD). However, the prognostic role of the PWR still needs to be investigated in LC patients with AD. In this study, we analyzed whether the PWR could stratify the risk of adverse outcomes (death or liver transplantation (LT)) in these patients. Methods: A prospective cohort of 1670 patients with AD of liver cirrhosis ((age: 55.2 ± 7.8, male = 1226 (73.4%)) was enrolled and evaluated for 28-day and overall adverse outcomes. Results: During a median follow-up of 8.0 months (range, 1.9–15.5 months), 424 (25.4%) patients had adverse outcomes (death = 377, LT = 47). The most common etiology of LC was alcohol use (69.7%). The adverse outcome rate was higher for patients with a PWR ≤ 12.1 than for those with a PWR > 12.1. A lower PWR level was a prognostic factor for 28-day adverse outcomes (PWR: hazard ratio 1.707, *p* = 0.034) when adjusted for the etiology of cirrhosis, infection, ACLF, and the MELD score. In the subgroup analysis, the PWR level stratified the risk of 28-day adverse outcomes regardless of the presence of ACLF or the main form of AD but not for those with bacterial infection. Conclusions: A lower PWR level was associated with 28-day adverse outcomes, indicating that the PWR level can be a useful and simple tool for stratifying the risk of 28-day adverse outcomes in LC patients with AD.

## 1. Introduction

Liver cirrhosis (LC) has been classified as compensated and decompensated. Compensated cirrhosis is usually known to have an asymptomatic course until increasing portal pressure and worsening liver function occur. These patients may have a good quality of life, and decompensated cirrhosis may progress undetected for several years. Decompensated cirrhosis is characterized by clinical signs of portal hypertension, such as ascites, esophagogastric varices, hepatic encephalopathy (HE), and jaundice. Following the first appearance of any of these signs, LC usually progresses more rapidly toward death or the need for liver transplantation (LT) [1]. Acute decompensation (AD) of cirrhosis and acute-on-chronic liver failure (ACLF) are two major challenges in chronic liver disease (CLD) patients, including those with LC. AD of cirrhosis is defined as the development of ascites, HE, jaundice, and/or gastrointestinal (GI) bleeding only in cirrhotic patients [2,3]. ACLF is defined as the failure of one or more major organs (the liver, kidneys, or brain, or the coagulation, circulatory, or respiratory systems) and always occurs in the setting of an episode of AD. Patients with ACLF have a high 28-day mortality after admission [4].

The pathophysiology of AD in cirrhotic patients or ACLF has been widely studied but is still unclear. Among the suggested and known hypotheses, systemic inflammation from bacterial infection and alcohol directly correlated with the severity of ACLF [5]. However, approximately 40–50% of ACLF patients have systemic inflammation without any identifiable precipitating triggers [2]. Systemic inflammation induces an impairment of the functions of one or more major organs and may be a common theme in the development of AD in cirrhotic patients [6].

The systemic inflammatory response is usually expressed by the levels of neutrophils, lymphocytes, platelets, and C-reactive protein (CRP). In the CANONIC study, a close relationship between the white blood cell count (WBC) and CRP and the presence and severity of ACLF was observed [2]. Recently, the neutrophil-to-lymphocyte ratio (NLR) and lymphocyte-to-monocyte ratio (LMR) have been used as indicators of the systematic inflammatory response and widely investigated as useful predictors of clinical outcomes in CLD patients [7,8,9,10]. In addition, the monocyte-to-lymphocyte ratio (MLR), platelet-to-lymphocyte ratio (PLR), red cell distribution width (RDW), RDW-to-platelet ratio, mean platelet volume, and mean corpuscular volume are prognostic factors and are associated with disease progression in hepatitis B virus (HBV)-related liver disease [11].

The platelet-to-white blood cell ratio (PWR) is a prognostic indicator for mortality in HBV-related ACLF and decompensated cirrhosis [12,13]. These studies demonstrated that the PWR predicted the prognosis similar to the Model for End-Stage Liver Disease (MELD) score for 30-day mortality. However, the prognostic role of the PWR in LC with other etiologies, such as alcohol use or hepatitis C virus infection, was not investigated. Therefore, we aimed to reveal whether the PWR could risk-stratify adverse outcomes (LT or death) in cirrhotic patients with AD.

## 2. Materials and Methods

### 2.1. Study Patients

The prospective Korean Acute-on-Chronic Liver Failure (KACLiF) study cohort consisted of patients who were hospitalized with acute deterioration of CLD, including either LC or noncirrhotic CLD, from July 2015 to August 2018. This study was approved by the Institutional Review Board of all 23 participating academic centers. In the KACLiF study, AD was defined as overt ascites, hepatic encephalopathy, GI bleeding, any kind of bacterial infection, and the deterioration of liver dysfunction, which was defined as a serum bilirubin level ≥3 mg/dL. LC was defined based on historical confirmation or when a cirrhotic configuration of the liver and/or splenomegaly was present in radiologic findings or when varices (abnormally enlarged veins, detected by upper endoscopy or cross-sectional images) and biochemical parameters were present [14]. We excluded patients who met any of the following exclusion criteria: (1) age < 18 years; (2) an absence of any chronic liver disease; (3) a presence of hepatocellular carcinoma; (4) a presence of severe chronic extrahepatic disease; (5) admission due to other chronic illness; (6) human immunodeficiency virus infection; (7) chronic decompensation of end-stage liver disease; (8) less than 28 days of follow-up, and (9) incomplete data. A total of 1773 patients were enrolled and followed up until July 2019. Among them, 1670 cirrhotic patients with AD were analyzed in this study.

### 2.2. Data Collection and the Definition of Clinical Parameters

We collected data about patient demographics, the etiology of liver disease, clinical and laboratory variables, types of AD, and the development of ACLF. The precipitating events included any kind of bacterial infection, GI bleeding, active alcoholism, a reactivation of viral hepatitis, toxic liver injury, and others. Active alcoholism was defined as more than 21 units/week in men and more than 14 units/week in women within 3 months prior to admission [3]. Systemic inflammatory response syndrome (SIRS) was followed by the criteria of the American College of Chest Physicians/Society of Critical Care Medicine [15]. The Child–Turcotte–Pugh (CTP) score, MELD score, serum sodium (Na) to MELD score (MELD-Na), MELD 3.0 [16], and chronic liver failure-sequential organ failure assessment (CLIF-SOFA) score were calculated based on the clinical variables within 24 h of admission. The patients who developed AD and organ failure were classified as having ACLF according to the CLIF-C definition. The PWR was defined as platelets divided by WBCs.

### 2.3. Primary Outcomes and Follow-Up

The primary endpoints of this study were 28-day and overall adverse outcomes during the follow-up period. Adverse outcomes were defined as death or LT. Person-years were censored on the date of death, LT, or the last date of follow-up, whichever came first. We evaluated where the PWR level was associated with 28-day and overall adverse outcomes. Subgroup analysis according to the etiology of cirrhosis, the main form of AD, and the presence of ACLF was performed to evaluate the effect of the PWR level on adverse outcomes.

### 2.4. Statistical Analysis

In all study subjects, variables were compared parametrically using Student’s t test, the Mann–Whitney U test, the *x*^2^-test, or Fisher’s exact test. To determine the cut off points of PWR, receiver operating characteristic (ROC) curve analysis were used. The 28-day and overall adverse outcomes were analyzed using the Kaplan–Meier method and compared with the log rank test. Cox proportional hazard regression analysis was performed to determine whether variables including the PWR level were associated with adverse outcomes. The association between the PWR level and adverse outcomes was tested in multivariable adjusted models with step-by-step inclusion of risk factors for adverse outcomes that showed an association in univariate analysis (*p* < 0.10). All reported *p* values were two-sided. Statistical significance was considered when the *p* value was less than 0.05. All statistical analyses were performed using SPSS V27.0 (SPSS, Inc., an IBM company, Chicago, IL, USA).

## 3. Results

### 3.1. Comparison of Baseline Characteristics

A total of 1773 consecutive patients were screened, of whom 1670 were enrolled. With a median follow-up of 8.0 months (interquartile range [IQR], 1.9–15.5 months), 424 (25.4%) cirrhotic patients had adverse outcomes (death = 377, LT = 47). The baseline characteristics of all patients are summarized in Table 1. Among the enrolled patients, 1226 patients were male (73.4%). The mean patient age was 55.2 years. The most common etiology of LC was alcohol use (*n* = 1164, 69.7%), followed by viral infection (*n* = 196, 11.7%), alcohol use and viral infection (*n* = 140, 8.4%), and others (*n* = 170, 10. 2%). The main features of AD were GI bleeding (32%) and ascites (28.9%). The median CPS was 8.0 (7.0–11.0), and the median MELD score was 17.0 (12.8–22.3). The median PWR of all patients was 13.5 (8.8–19.8). The patients with a PWR ≤ 12.1 were younger and showed more active alcoholism at admission than the patients with a PWR > 12.1. Patients with a low PWR had higher CPS, MELD, and CLIF-SOFA scores than those with a high PWR. The 28-day and overall adverse outcomes were significantly worse in the patients with a low PWR than in the patients with a high PWR (*p* < 0.001).

### 3.2. Associated Factors for 28-Day and Overall Adverse Outcomes of AD of Liver Cirrhosis

**Figure 1:**
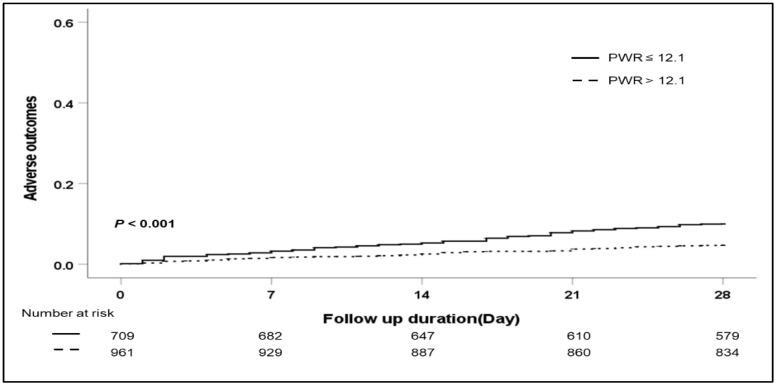
Cumulative 28-day adverse outcomes according to the PWR level. Abbreviation: PWR, platelet-to-white blood cell ratio.

The association between the PWR level and the 28-day adverse outcomes is shown in Table 2. In univariate analysis, bacterial infection, the etiology of cirrhosis, bilirubin, albumin, INR, sodium, PWR level, ACLF, and MELD score were significant factors for 28-day adverse outcomes. In multivariable analysis, a low PWR was a significant factor for 28-day adverse outcomes (hazard ratio (HR) 1.707, *p* = 0.034). The presence of ACLF and a high MELD score were significant risk factors for short-term adverse outcomes (HR 1.729, *p* = 0.045; HR 1.101, *p* < 0.001). During the 28-day follow-up, 111 patients died. The development of adverse outcomes was significantly higher in patients with low PWR levels than in patients with high PWR levels (Figure 1) (*p* < 0.001). However, when further evaluating the relationship between the PWR level and the overall adverse outcomes, multivariate analysis demonstrated that the MELD score was the only significant risk factor for overall adverse outcomes (Table 3).

### 3.3. Subgroup Analysis: Effects of the PWR Level on the 28-Day Adverse Outcomes According to the Etiology of Cirrhosis and the Type of AD

**Figure 2:**
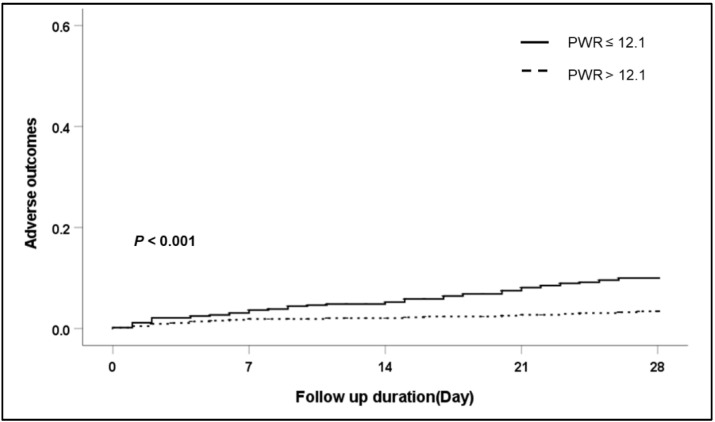
Cumulative 28-day adverse outcome in patients with alcohol liver cirrhosis according to PWR level. Abbreviation: PWR, platelet-to-white blood cell ratio.

To confirm the association of the PWR level and 28-day adverse outcomes in detail, we performed subgroup analysis according to the etiology of cirrhosis and the main type of AD (Table 4). Regardless of the etiology of cirrhosis (viral infection, alcohol use, viral infection and alcohol use, and other), patients with a low PWR showed an increased risk of 28-day adverse outcomes compared with patients with a high PWR. Especially in patients with alcoholic cirrhosis, those with a low PWR level presented significantly increased adverse outcomes compared with those with a high PWR level (*p* < 0.001). (Figure 2). In a subgroup analysis according to the main type of AD (ascites, bacterial infection, GI bleeding, HE, and jaundice), a low PWR level was more associated with the development of 28-day adverse outcomes than a high PWR level regardless of the type of AD except bacterial infection. Among patients with AD caused by bacterial infection, there was no significant difference in 28-day adverse outcomes according to the PWR level (*p* = 0.524).

### 3.4. Subgroup Analysis: Effects of the PWR Level on the 28-Day Adverse Outcomes According to the Presence of ACLF

**Figure 3:**
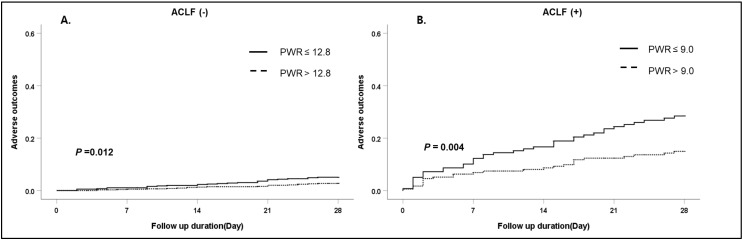
Cumulative 28-day adverse outcomes according to ACLF. (**A**) Absence of ACLF, (**B**) presence of ACLF. Abbreviation: ACLF, acute-on-chronic liver failure; PWR, platelet-to-white blood cell ratio.

Among 1670 cirrhotic patients with AD, 315 (18.9%) developed ACLF at admission according to the CLIF-C definition (Table 1). The patients who had ACLF at admission demonstrated a poor adverse outcome compared with patients who did not have ACLF. (63/315 (20%) vs. 48/1355 (3.5%), *p* = 0.001)). The median PWR level of both the absence of ACLF and presence of ACLF were 14.1 (9.7–20.5) and 9.9 (6.4–15.3), respectively. In both groups, the development of adverse outcomes was significantly higher in patients with low PWR levels than in patients with high PWR levels (Figure 3) (absence of ACLF, *p* = 0.012; presence of ACLF, *p* = 0.004).

## 4. Discussion

This study revealed that a low PWR level was a prognostic factor associated with 28-day adverse outcomes (death or LT) in cirrhotic patients with AD. Low PWR levels correlated with adverse outcomes even after adjusting for well-known prognostic factors for mortality in LC, such as the presence of ACLF and the MELD score. A low PWR tended to increase the risk of adverse outcomes compared with a high PWR level regardless of the etiology of cirrhosis.

Systemic inflammation in decompensated cirrhosis is a chronic condition related to products of bacterial translocation from the gut to the systemic circulation. Episodic aggravations of bacterial translocation or proinflammatory precipitants are related to the development of one or more organ failures [6]. AD of cirrhosis caused by bacterial infection, GI bleeding, HBV reactivation, or the use of hepatotoxic drugs is the consequence of a broad spectrum of activated inflammatory cytokine pathways [5,17]. Rolando et al. revealed that 887 patients with acute liver failure were investigated by sequential assessment of SIRS, which was present in 56% of patients irrespective of whether the patients had bacterial infections. The severity of SIRS was associated with a more critical illness, the progression of encephalopathy, organ failure, and death [18].

Inflammatory biomarkers, such as the WBC, neutrophil, lymphocyte, and platelet counts and acute phase reactants, are easily available, relatively simple, and popular in clinical settings. The combination of these markers, such as the NLR, MLR, and PLR, has been studied for prognostic value in a variety of liver diseases. Interestingly, the NLR has already been identified as a short- and long-term prognostic marker for patients on the liver transplant waiting list and hospitalized patients with cirrhosis, ACLF, and AD without ACLF [10,19,20,21].

In terms of the importance of the PWR in this study, the role of thrombocytopenia in patients with CLD has several mechanisms, such as a reduction in hepatic thrombopoietin production, splenomegaly due to increased splenic vein pressure, immune-mediated destruction, and destruction resulting from an inflammatory response with tumor necrosis factor [22,23]. Thus, a low platelet count is closely associated with the severity of LC. The WBC count, especially neutrophils, reflects ongoing inflammation. The systemic inflammatory response associated with endotoxemia induced an increased serum neutrophil count [10,24]. Circulating neutrophils exhibit impaired phagocytic function and bactericidal capacity, which are predictors of mortality in LC [25]. Previous studies in cirrhotic patients associated with HBV demonstrated that a low PWR level was a poor prognostic marker in decompensated cirrhosis and ACLF [12,13]. Along with these results, our finding is in line with previous clinical reports showing an association between a low PWR and adverse outcomes of liver disease not only in viral hepatitis but also in other etiologies of cirrhosis. In this study, the association of a low PWR level and adverse outcomes was statistically significant regardless of the clinical types of AD in cirrhotic patients except bacterial infection. In a previous prospective study of cirrhotic patients hospitalized for bacterial infection, the mean WBC count was significantly increased in cirrhotic patients with bacterial infection compared with cirrhotic patients without infection. However, most of them had WBC counts within the normal range due to leukopenia with splenomegaly [26]. Our results also showed that patients with bacterial infection had higher WBC counts than patients without bacterial infection at admission (*p* < 0.001). Additionally, low platelet counts were observed in patients with infection (*p* < 0.001). As a result, the PWR level was further affected by bacterial infection and inflammation, along with the effect of hepatic dysfunction in AD, and its level was reduced compared to other clinical types of AD that did not directly affect the PWR level. Recently, a prospective multicenter cohort named the Chinese Acute-on-Chronic Liver Failure (CATCH-LIFE) study, which enrolled 3970 patients with acute-on-chronic liver disease, reported that lower platelet counts increased the risk of 90-day adverse outcomes (death or LT) in these patients. They focused on the platelet count level and included patients with and without cirrhosis and patients with and without ACLF. However, patients with lower platelet count levels showed poor adverse outcomes regardless of cirrhosis but not ACLF [27]. ACLF is a well-known poor prognosis factor. ACLF predicts short-term and long-term mortality and is a better predictive factor than the MELD and CPS scores in AD with CLD [2]. In our study, ACLF was an important prognostic factor for 28-day adverse outcomes in cirrhotic patients with AD when combined with the MELD score and PWR level (Table 2). In the subgroup analysis, a low PWR level was associated with increased adverse outcomes regardless of whether patients had ACLF (Figure 3). This finding suggests that the PWR level is a more useful prognostic biomarker regardless of the severity of AD in cirrhotic patients than the platelet level alone. In Asia, HBV (76%) is a major cause of ACLF [3,28], which is associated with increased development of liver and coagulation failure. Currently, the APASL definition does not include the status of cirrhosis. However, the KACLiF study involving 1470 prospectively enrolled cirrhotic patients revealed that the major etiology was alcohol use (72%) [29]. ACLF generally occurs among patients with alcoholic cirrhosis (60%) and is caused by infection, alcohol use, or both in Western countries [2]. Therefore, we suggest that the PWR might be a simple and good surrogate marker for the risk of adverse outcomes in these patients regardless of etiology.

This study has some limitations. First, alcohol use was the main etiology of cirrhosis and acute insults in this study. A previous retrospective multicenter study in Korea also found that the main cause of CLD with AD was alcohol use [30]. This is considered to be the result of the widespread implementation of a universal HBV vaccination program and oral antiviral therapy for HBV infection in Korea, which has been recommended since 2012 [31]. In addition, Korean culture is tolerant of drinking, and unlike in other Asian countries, alcohol-related liver disease rather than liver disease due to other etiologies is a major issue. Second, the long-term adverse outcomes could not be accurately identified because the follow-up period was not long, so the PWR did not obtain any meaningful results for the long-term prognosis.

Nonetheless, our study also has several strengths, including the large number of patients with complete data in prospective study settings. This is the first prospective study to evaluate whether the PWR level, a simple and easily tractable hematologic marker, can predict adverse outcomes according to the various etiologies and clinical forms in AD patients with cirrhosis. Additionally, PWR levels could stratify the risk of adverse events in AD patients who may or may not have ACLF.

## 5. Conclusions

In conclusion, a low platelet-to-white blood cell ratio was associated with 28-day adverse outcomes in patients with acute decompensation with cirrhosis, along with ACLF and the MELD score. This simple hematologic parameter might be a good surrogate marker for the risk of adverse outcomes in these patients regardless of etiology and clinical forms.

## Figures and Tables

**Table 1:** Baseline characteristics according to the PWR level.

	ALL (*n* = 1670)	PWR ≤ 12.1 (*n* = 709)	PWR > 12.1 (*n* = 961)	*p* Value
Age (years)	55.2 ± 11.4	53.4 ± 10.9	56.5 ± 11.6	<0.001
Male (%)	1226 (73.4)	535 (75.5)	691 (71.9)	0.104
Etiology of cirrhosis, *n* (%)				<0.001
Viral infection (HBV/HCV)	196 (163/33, 11.7)	70 (56/14, 9.8)	126 (107/19, 13.1)	
Alcohol use	1164 (69.7)	552 (73.6)	642 (66.8)	
Viral infection + alcohol use	140 (8.4)	68 (9.6)	72 (7.5)	
Others +	170 (10.2)	49 (6.9)	121 (12.6)	
Acute decompensation, *n* (%)				
Ascites	483 (28.9)	181 (25.5)	302 (31.4)	0.009
Bacterial infection	172 (10.3)	105 (14.8)	67 (7.0)	<0.001
SBP	27 (1.6)	20 (2.1)	7 (1.0)	
Intra-abdominal infection	17 (1.0)	12 (1.2)	5 (0.7)	
Pneumonia	12 (0.7)	8 (0.8)	4 (0.6)	
Urinary tract infection	14 (0.8)	13 (1.3)	1 (0.1)	
Skin infection	6 (0.4)	5 (0.5)	1 (0.1)	
Unknown origin	96 (5.7)	70 (7.2)	26 (3.7)	
GI bleeding	534 (32)	252 (35.5)	282 (29.3)	0.007
HE	274 (16.4)	112 (15.8)	162 (16.9)	0.563
Jaundice	491 (29.4)	232 (32.7)	259 (27.0)	0.011
CVD, *n* (%)	70 (4.2)	27 (3.8)	43 (4.5)	0.502
DM, *n* (%)	458 (27.4)	180 (25.4)	278 (28.9)	0.109
HTN, *n* (%)	353 (21.1)	130 (18.3)	223 (23.2)	0.016
Precipitating events, *n* (%)				
Active alcoholism	748 (44.8)	354 (49.9)	394 (41.0)	<0.001
Bacterial infection	134 (8.0)	85 (12.0)	49 (5.1)	<0.001
GI bleeding	474 (28.4)	230 (32.4)	244 (25.4)	0.002
Viral reactivation	31 (1.9)	8 (1.1)	23 (2.4)	0.058
Toxic	19 (1.1)	4 (0.6)	15 (1.6)	0.058
Others	88 (5.3)	25 (3.5)	63 (6.6)	0.006
ACLF, *n* (%)	315 (18.9)	196 (27.6)	119 (12.4)	<0.001
SIRS, *n* (%)	411 (24.6)	239 (33.7)	172 (17.9)	<0.001
HCC, *n* (%)	76 (4.6)	27 (3.9)	49 (5.2)	0.206
PWR	13.5 (8.8–19.8)	8.2 (6.2–10.1)	18.6 (14.7–25.1)	<0.001
PNR	21.3 (12.8–36.9)	11.7 (8.2–15.9)	31.4 (22.8–49.4)	<0.001
Laboratory data				
WBC count ×10^9^/L	8.21 (4.75–10.10)	9.14 (6.56–13.10)	5.75 (4.09–7.92)	<0.001
Hemoglobin, g/dL	10.3 (8.4–12.1)	10.0 (8.2–11.9)	10.5 (8.5–12.2)	0.015
Platelet, mg/L	94 (63–137)	69 (48–99.5)	114 (82–161)	<0.001
Bilirubin mg/dL	3.3 (1.5–7.7)	4.7 (2.3–9.9)	2.4 (1.1–5.5)	<0.001
Albumin, g/dL	2.8 (2.5–3.3)	2.7 (2.4–3.1)	2.9 (2.6–3.4)	<0.001
INR	1.5 (1.3–1.8)	1.6 (1.4–2.0)	1.4 (1.2–1.7)	<0.001
Creatinine, mg/dL	0.9 (0.7–1.3)	0.9 (0.7–1.5)	0.9 (0.7–1.1)	<0.001
Sodium, mEq/L	136 (132–139)	135 (130–139)	136 (133–140)	<0.001
Child–Pugh score	8.0 (7.0–11.0)	10.0 (8.0–11.0)	9.0 (7.0–10.0)	<0.001
MELD score	17.0 (12.8–22.3)	19.6 (15.3–25.4)	15.2 (11.2–19.7)	<0.001
MELD-Na score	19.9 (14.8–26.0)	23.4 (17.3–29.0)	18.1 (13.1–23.2)	<0.001
MELD-3 score	16.9 (10.0–23.8)	20.5 (13.3–27.6)	14.6 (8.3–20.7)	<0.001
CLIF-SOFA score	5.0 (4.0–7.0)	6.0 (5.0–8.0)	5.0 (3.0–6.0)	<0.001
Adverse outcome, *n* (%)				
28-day follow-up	111 (6.6)	68 (9.6)	43 (4.5)	<0.001
LT	12 (0.7)	7 (1.0)	5 (0.5)	
Overall follow-up	424 (25.4)	220 (31.0)	204 (21.2)	<0.001
LT	47 (2.8)	22 (3.1)	25 (2.6)	

Abbreviation: HBV, hepatitis B virus; HCV, hepatitis C virus; GI, gastrointestinal; SBP, spontaneous bacterial peritonitis; CVD, cardiovascular disease; ACLF, acute-on-chronic liver failure; SIRS, systemic inflammatory response syndrome; HCC, hepatocellular carcinoma; PWR, platelet-to-white blood cell ratio; PNR, platelet-to-neutrophil ratio; WBC, white blood cell; INR, international normalized ratio; MELD, Model for End-Stage Liver Disease; CLIF-SOFA, Chronic Liver Failure-Sequential Organ Failure Assessment; LT, liver transplantation. + others etiology of cirrhosis included cryptogenic (*n* = 122), autoimmune hepatitis (*n* = 37), and primary biliary cirrhosis (*n* = 11).

**Table 2:** Risk factors for 28-day adverse outcomes of AD of liver cirrhosis.

	Univariate HR (95% CI)	*p* Value	PWR	
			Multivariate HR	*p* Value
Age	1.003 (0.984–1.020)	0.691		
Sex	1.099 (0.716–1.686)	0.667		
Cirrhosis etiology	Ref. (others)		Ref. (others)	
Viral infection	2.835 (1.038–7.738)	0.042	2.169 (0.787–5.981)	0.135
Alcohol use	2.175 (0.878–5.386)	0.093	1.155 (0.459–2.905)	0.759
Viral infection and alcohol use	4.985 (1.861–13.35)	0.001	2.407 (0.884–6.557)	0.086
Active alcoholism	1.140 (0.786–1.655)	0.490		
Bacterial infection	2.118 (1.264–3.551)	0.004	1.156 (0.676–1.976)	0.596
GI bleeding	0.847 (0.552–1.300)	0.488		
Toxic	0.806 (0.113–5.774)	0.830		
Bilirubin	1.072 (1.056–1.089)	<0.001		
Albumin	0.294 (0.207–0.418)	<0.001		
INR	1.315 (1.242–1.392)	<0.001		
Na	0.944 (0.920–0.968)	<0.001		
PWR	3.157 (1.963–5.077)	<0.001	1.707 (1.042–2.975)	0.034
ACLF	6.374 (4.378–9.281)	<0.001	1.729 (1.013–2.950)	0.045
HCC	0.569 (0.181–1.792)	0.335		
MELD score	1.130 (1.110–1.150)	<0.001	1.101 (1.072–1.131)	<0.001

Abbreviations: HR, hazard ratio; PWR, platelet-to-white blood cell ratio; GI, gastrointestinal; INR, international normalized ratio; ACLF, acute-on-chronic liver failure; MELD, Model for End-Stage Liver Disease; HCC, hepatocellular carcinoma.

**Table 3:** Risk factors for overall adverse outcomes of AD of liver cirrhosis.

	Univariate HR (95% CI)	*p* Value	PWR	
			Multivariate HR	*p* Value
Age	1.003 (0.995–1.0101)	0.490		
Sex	1.024 (0.824–1.271)	0.833		
Cirrhosis etiology	Ref. (others)		Ref. (others)	
Viral infection	1.003 (0.652–1.544)	0.989		
Alcohol use	1.191 (0.853–1.664)	0.304		
Viral infection and alcohol use	1.670 (1.092–2.577)	0.018		
Active alcoholism	1.098 (0.907–1.330)	0.337		
Bacterial infection	1.641 (1.201–2.244)	0.002	1.051 (0.759–1.455)	0.771
GI bleeding	0.670 (0.535–0.839)	<0.001	0.841 (0.666–1.063)	0.143
Toxic	0.412 (0.103–1.654)	0.211		
Bilirubin	1.052 (1.042–1.063)	<0.001		
Albumin	0.420 (0.353–0.499)	<0.001		
INR	1.357 (1.297–1.419)	<0.001		
Na	0.941 (0.928–0.953)	<0.001		
PWR	0.610 (0.498–0.748)	<0.001	0.852 (0.687–1.056)	0.413
ACLF	2.863 (2.333–3.515)	<0.001	1.177 (0.881–1.571)	0.270
HCC	0.853 (0.544–1.338)	0.489		
MELD score	1.088 (1.076–1.100)	<0.001	1.088 (1.076–1.100)	<0.001

**Table 4:** Effect of PWR level on 28-day adverse outcomes according to subgroup analysis.

	PWR ≤ 12.1		PWR > 12.1		
	Total	Event	Total	Event	*p* Value
Etiology of cirrhosis					
Viral infection	70	7	126	9	0.519
Alcohol use	522	50	642	21	<0.001
Viral infection + alcohol use	68	7	72	12	0.282
Others	49	4	121	1	0.010
Acute decompensation					
Ascites					
No	528	48	659	31	0.002
Yes	181	20	302	12	0.003
Infection					
No	604	57	894	34	<0.001
Yes	105	11	67	9	0.524
GI bleeding					
No	403	45	627	33	<0.001
Yes	306	23	334	10	0.011
HE					
No	597	50	799	33	0.001
Yes	112	18	162	10	0.008
Jaundice					
No	477	35	702	30	0.027
Yes	232	33	259	13	<0.001

Abbreviations: PWR, platelet-to-white blood cell ratio; GI, gastrointestinal; HE, hepatic encephalopathy.
